# Assessing climate change preparedness in hospitals and nursing homes in Hesse, Germany

**DOI:** 10.1016/j.joclim.2026.100685

**Published:** 2026-04-14

**Authors:** Caroline Maria Körner, Max Geraedts

**Affiliations:** Institute for Health Services Research and Clinical Epidemiology, Marburg University, Marburg, Hesse, Germany

**Keywords:** Climate resilience, Hospitals, Nursing homes, Climate mitigation, Climate adaptation, Heat protection

## Abstract

**Introduction:**

Vulnerable groups are particularly affected by climate change. The healthcare sector is responsible for approximately 6% of the greenhouse gas emissions in Germany. It remains unclear to what extent hospitals and nursing homes in Germany have already implemented climate mitigation and adaptation measures.

**Materials and methods:**

The study was conducted in the predominantly rural Marburg-Fulda region of Hesse, Germany, which has a population of 2,028,359. A total of 91 of the 300 nursing homes (30%) and seven of the 32 hospitals (22%) from this region participated in a semi-standardized telephone survey. Data were collected between January 28, 2025, and July 31, 2025. Data were first analyzed descriptively. In the next step, the Fisher‒Freeman‒Halton test was used to identify correlations between institutional characteristics and various climate mitigation and adaptation measures.

**Results:**

Nursing home size was positively correlated with the presence of climate mitigation guidelines (*p* = 0.012). Simple climate mitigation measures for everyday use (e.g., switching off lights or lowering heating) are generally implemented in nursing homes and hospitals, whereas larger, cost-intensive climate protection measures are less frequently implemented. Except for heat protection, climate adaptation measures are not very widespread. Barriers to the implementation of measures include financial constraints, staffing shortages, and structural limitations.

**Conclusion:**

Hospitals and nursing homes in the Marburg–Fulda region are not yet climate-resilient. Implementing such measures often fails because of a lack of resources. To create a climate-resilient healthcare system, targeted financial and structural support is needed from policymakers.

## 1 Introduction

Climate change is considered among the greatest health threats of the 21st century. As early as 2009, the World Health Organization (WHO) focused on the direct and indirect effects of climate change on human health [1]. In 2023, the Robert Koch Institute (RKI) conducted a risk analysis, which summarized the most significant climate-related health risks for Germany: heat stress, air pollution, allergic reactions and extreme weather events [1,2]. Statistics show that heat-related mortality is a widespread worldwide problem. The number of heat-related deaths among people older than 65 years has increased by more than 50% in the last 20 years [3].

Older people with weakened general health and impaired thermoregulation [1] as well as people with preexisting cardiovascular conditions are particularly negatively affected by climate change [2]. This growing vulnerability among certain population groups not only increases individual health risks but also places additional strain on healthcare systems. The European heatwave of 2003, which was responsible for more than 70,000 additional deaths, revealed that some of these deaths were also due to bottlenecks in the healthcare system [4].

Moreover, the healthcare sector itself contributes to climate change. It accounts for approximately 6% of Germany’s total greenhouse gas emissions [5]. Although many hospitals express the intention to engage in climate mitigation, there are significant differences in the actual organization and implementation of related measures [6]. International research has further indicated that healthcare providers are not sufficiently prepared for the effects of climate change [7]. A 2022 survey by the German Hospital Institute revealed that only 38% of the hospitals had guidelines or targets related to climate protection [8]. In particular, larger institutions are more likely to have structured responsibilities [6].

Existing studies have shown that climate mitigation activities in hospitals are diverse. Energy-saving measures are frequently implemented, while more cost-intensive measures often remain unaddressed [8]. In contrast to mitigation, climate adaptation in healthcare primarily aims to reduce the health risks associated with the increasing frequency and intensity of extreme weather events due to climate change, particularly heat events, as defined by the Intergovernmental Panel on Climate Change (IPCC). In this context, heat protection measures can be understood as a central element of climate adaptation [9]. Various projects across Germany and Europe aim to promote climate mitigation and adaptation in inpatient facilities—for example, the “Green Hospitals”, “KLiK green” and “klimafreundlich pflegen” initiatives [[10], [11], [12]]. The state of Hesse (Germany) has already carried out extensive preparatory work, for example, in the form of a heat health action plan [13].

International studies have already shown that extreme weather events lead to increased hospitalization rates and worsening disease symptoms [14,15]. People with multiple morbidities are particularly at a high risk of mortality in hot weather [16]. While institutional care structures could be adapted to such conditions—for example, by increasing staffing during heatwaves—these adaptive strategies are rarely reported in practice.

Despite the growing recognition of the challenges posed by climate change, there is still limited knowledge about how climate mitigation and adaptation are embedded within everyday operations in healthcare settings. To address this gap, the present study—funded as part of the Hessian LOEWE research project “HABITAT—Health Affected by Climate Change and Air Pollution—Pathophysiology and Regional Management” (LOEWE/2/519/03/09.001(0005)/99)—examines the implementation of climate mitigation and climate adaptation measures in hospitals and nursing homes in a predominantly rural region of Hesse, Germany. The primary research question analyzes how hospitals and nursing homes are prepared for climate change and to what extent they have already taken adaptation or mitigation actions.

The following hypotheses (**H**) are examined in detail:

H1 The larger the hospital or nursing home provider is, the more likely the facility is to have

a. guidelines on climate mitigation
b. guidelines on climate adaptation.

H2 Less than half of the hospitals and nursing homes have implemented measures

a. for climate mitigation
b. for climate adaptation

that go beyond basic measures such as energy savings (climate mitigation) and heat protection (climate adaptation).

H3 The greater the number of beds in a hospital or nursing home, the more frequently it implements measures

a. on climate mitigation
b. on climate adaptation.

H4 The larger the hospital or nursing home provider, the more frequently it implements measures

a. for climate mitigation
b. for climate adaptation.

H5 Less than half of hospitals and nursing homes consider climate adaptation measures that affect staffing arrangements, such as modifying work schedules or staff allocation during periods of heat.

## 2 Methods

### 2.1 Data collection and measures

The study region covers the districts of Marburg-Biedenkopf and Fulda and their surrounding districts in the federal state of Hesse, Germany, with a total population of 2,028,359. A total of 32 hospitals and 300 nursing homes were identified in the region on the basis of data from quality reports (§ 136b (1) sentence 1 no 3 SGB V), online research, and subsequent manual research [17].

The data were collected via telephone survey. Following initial written contact, the hospitals and nursing homes were contacted by telephone. A total of 734 calls were made to nursing homes to establish contact and conduct the survey, with each nursing home called up to seven times. A total of 56 calls were made to hospitals, with each hospital contacted up to five times.

The survey was conducted between January 28 and July 31, 2025 as a personal, oral semi-standardized telephone interview lasting five to ten minutes [18]. Telephone interviews were conducted with the management or director of the facility. In rare cases, the management delegated participation to a person responsible for the topic (e.g., technical service, nursing service management). The survey instruments differed slightly for hospitals and nursing homes (see Supplementary Material). The following subjects were identified as being relevant to hospitals and nursing homes:

− Existence of climate mitigation and/or climate adaptation guidelines
− Strategies for climate mitigation
− Strategies for climate adaptation
− Barriers and need for support

Furthermore, data from heat protection tests conducted by the Hessian State Office for Health and Care (HLfGP) were evaluated for care facilities. The lead author of the study contacted the HLfGP, which voluntarily provided these data.

### 2.2 Sample

For 91 of the 300 nursing homes, management or senior staff members participated in the survey (participation rate of 30%). The study included only facilities specializing in the care of older people. The majority of facilities are located in rural areas; only two are classified as being in ‘urban or densely populated areas’ according to the spatial classification system of the Federal Institute for Research on Building, Urban Affairs and Spatial Development (BBSR) [19]. According to the Hessian Agency for Nature Conservation, Environment and Geology (HLNUG), 77% of the locations are in regions with a high heavy rainfall index [20]. Furthermore, the HLNUG published the average heat stress for Hesse for the summer months from 2001 to 2020. The average temperatures over the 20-year period indicate that 18% of the facilities are located in ‘warm’ areas, 54% in ‘very warm’ areas, and 11% in ‘hot’ areas [21].

Seven of the 32 hospitals participated in the survey (participation rate 22%). Owing to the small sample size and the associated possibility of identification, no geographical details can be provided.

### 2.3 Data analysis

The lead author was responsible for categorizing the responses to the open-ended questions. The resulting variables, their characteristics and more detailed information about what the institutions responded to are listed in the Supplementary Material. First, the data were evaluated descriptively. To test hypotheses H1, H3 and H4, group differences were examined (e.g., by the number of beds or providers). Owing to the small sample size for the analyses (expected frequencies<5) of the nursing facilities, the Fisher–Freeman–Halton test was used. The hospital data could only be evaluated descriptively. All the analyses were performed using SPSS version 30.0.0.

### 2.4 Ethics

This study was approved by the Ethics Committee of Marburg University (24–305 BO). All the participants were informed about the procedure and data analysis prior to the survey and gave their consent. The data collected were pseudonymized after the telephone survey.

## 3 Results

### 3.1 Characteristics of the facilities

The seven participating hospitals have a median number of 354 beds, and the annual case numbers range from approximately 6,800 to 44,000 cases per year, with a median of 11,457 cases per year. The participating nursing homes have between 11 and 254 beds, with a median of 69 beds. In terms of provider size, the range extends from single-owner facilities to large associations with up to 300 facilities. Across all participating providers, the median number of nursing homes operated per provider is 4.5.

### 3.2 Guidelines

29% of the hospitals reported having policies to mitigate climate change; 43% reported having policies to adapt to climate change.

**Fig. 1:**
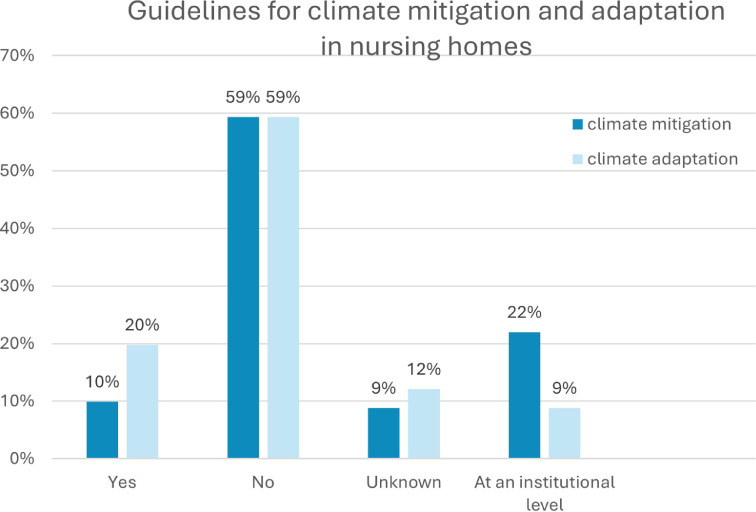
Guidelines for climate mitigation and adaptation in nursing homes.

The data according to the guidelines in nursing homes are shown in Fig. 1.

Nursing homes with climate mitigation guidelines were mostly owned by providers with at least two facilities. No single-owner facility had its own climate mitigation guidelines. The size of the nursing home and the presence of climate mitigation guidelines were significantly correlated (*p = 0.012*). According to Cramer’s V, the effect size was 0.346, indicating a moderate to strong practical correlation. An evaluation of the adjusted standardized residuals clearly revealed that smaller providers are less likely to have climate mitigation guidelines (residual = −1.9), whereas larger providers are more likely to have them (residual = +1.9). These residuals were marginally below the conventional significance threshold of ± 1.96. The combination of the Fisher–Freeman–Halton test and the residual analysis thus provided confirmation of H1a. Owing to the small sample size, it was not possible to analyze this association among hospitals.

No statistically significant correlation was observed between climate adaptation guidelines and either provider size or number of beds. H1b was rejected for nursing homes, and an analysis for hospitals could not be conducted.

### 3.3 Implementation of climate mitigation measures

All hospitals surveyed stated that they implement climate mitigation measures; 86% consistently implemented energy-saving measures. These often consist of various individual measures, such as turning off lights, lowering heating, switching to LED lighting or changing electricity providers. A total of 43% of the hospitals reported implementing measures to reduce waste. In addition, individual hospitals are implementing various measures, such as digitization to reduce paper use and printing and menu adjustments (e.g., regional sourcing and reduced meat content) to decrease food-related greenhouse gas emissions. Almost 60% of the hospitals reported switching to more environmentally friendly anesthetic gases.

**Fig. 2:**
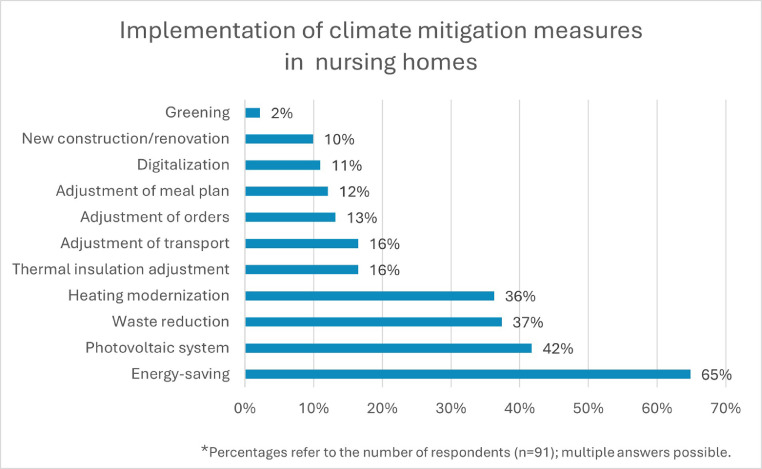
Implementation of climate mitigation measures in nursing homes.

90% of the nursing homes surveyed reported that they are implementing climate mitigation measures. As shown in Fig. 2, the range of individual measures is wide.

Therefore, H2a can be accepted for nursing homes and hospitals.

For nursing homes, no significant correlation was observed between provider size or number of beds and the implementation of climate mitigation measures (rejection of H3a and H4a). An analysis could not be conducted for hospitals.

### 3.4 Implementation of climate adaptation measures

One hospital reported that it is implementing climate adaptation measures and described how it is prepared for storms and flooding. The remaining six participating hospitals stated that they are implementing only heat protection measures.

Among the 91 nursing homes, 74 (81%) reported having implemented measures to adapt to climate change. The facilities primarily understood climate adaptation as heat protection. Three nursing homes stated that they protect themselves against storms, while 23 facilities (25%) stated that they protect themselves against heavy rain and/or flooding. However, no correlation was observed between an increased heavy rainfall index and the implementation of protective measures against heavy rain/flooding.

H2b can be accepted, as heat protection plays a dominant role in both types of facilities.

H3b and H4b are rejected for nursing homes because no correlation was observed between provider size, number of beds, and the implementation of climate adaptation measures. An analysis could not be conducted for hospitals.

### 3.5 Heat protection

All the nursing homes and hospitals stated that they implemented heat protection measures in accordance with official requirements. In total, 45% of the nursing homes had air conditioning in the communal areas for residents and/or in the staff rooms. In hospitals, air conditioning was generally limited to operating rooms and intensive care units. No facility could guarantee complete air conditioning throughout the building.

Of the nursing homes, 37% stated that they could adapt staffing arrangements for employees in hot weather, for example, by changing working hours or adjusting the number of staff on duty. However, 60% of the nursing homes stated that this is not a common practice. Only two of the seven hospitals reported the possibility of adjusting schedules for medical and nursing staff in hot weather. H5 can be assumed, as only a small proportion of facilities consider adapting staffing or supply structures for heat protection.

An evaluation of regular, random heat protection tests conducted by the HLfGP revealed that almost all the nursing homes were implementing individual heat protection measures. Measures included subscribing to the German Meteorological Service newsletter, informing employees about heatwaves, and ensuring that medication is stored within the prescribed temperature limits.

### 3.6 Barriers and need for support

**Fig. 3:**
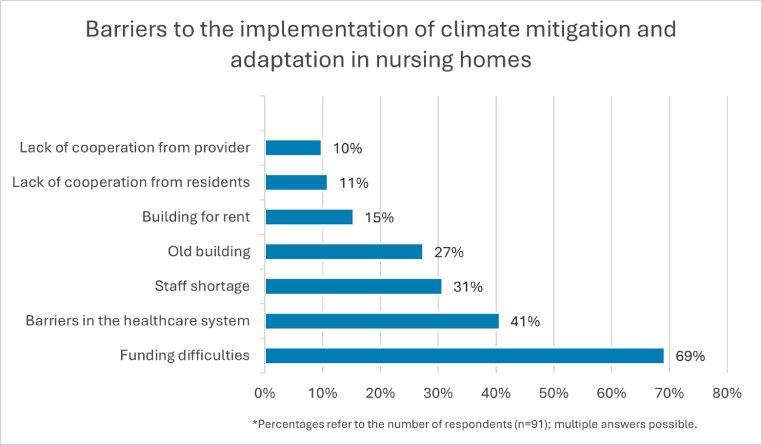
Barriers to the implementation of climate mitigation and adaptation in nursing homes.

Both hospitals and nursing homes encounter challenges in implementing climate measures. All hospitals reported financial constraints. Furthermore, hospitals indicated that the German healthcare system is not designed to cope with the consequences of climate change. All of the challenges faced by nursing homes are summarized in Fig. 3.

57% of the nursing homes and all the participating hospitals stated that they needed more financial support. Almost one-third of the nursing homes and more than 85% of the hospitals expressed a desire for greater political support. The facilities indicated that legal obligations for retrofitting, accompanied by financial incentives, would be helpful.

## 4 Discussion

The results of this study revealed that most hospitals and nursing homes lack climate mitigation or adaptation policies. Implemented measures are generally low-cost. Heat protection measures have been implemented, but they rarely include solutions such as installation of air conditioning systems. Key barriers to implementation include financial constraints, insufficient political support, and staffing shortages.

In accordance with climate mitigation or adaptation guidelines, our study confirms the findings of Schmidt et al., who reported analogous findings for hospitals. According to their findings, approximately 32% of the institutions surveyed demonstrate a lack of strategic planning for climate mitigation [6]. By contrast, Janson et al. reported that all six nursing homes in Hesse that participated in their survey had a heat protection policy in place [22]. In our study, which explicitly inquired about guidelines for climate change adaptation, with heat protection being identified as a sub-area, only one-fifth of the respondents confirmed the presence of such guidelines. Owing to the limited sample size in Janson et al.’s study and our own survey, making any conclusive, generalized statements is impossible [22]. It is plausible that the respondents may be unfamiliar with this concept or that it is not available in a written form.

Hypothesis H1a was confirmed for the nursing homes, that is, smaller providers are less likely to have climate mitigation guidelines than larger providers are. However, this does not apply to guidelines for climate adaptation in the Marburg–Fulda region; thus, H1b was rejected. Although there is no relevant literature specifically referring to the circumstances in nursing homes, evidence can be derived from the hospital sector. Larger clinics, such as university hospitals, are more likely to have effective climate mitigation strategies, which confirms our findings [6]. The literature suggests that climate adaptation strategies are often less formalized, as they are typically short-term, situation-dependent and practice-oriented. Their implementation depends more on individual awareness [23]. These differences could explain the difference between the mitigation and adaptation guidelines.

Energy-saving measures are most frequently cited as practical climate mitigation measures implemented in facilities. Larger measures are often not feasible for cost reasons, which confirms H2a. Previous surveys in German hospitals validate this finding [8].

Heat protection is consistently implemented in all facilities as a climate adaptation measure (H2b). A recent study in Hesse on heat protection revealed that this is well integrated into everyday care and heat protection measures are implemented in accordance with official guidelines [22]. Similarly, the detailed Hessian heat health action plan and the HLfGP’s recommendations for action during exceptional heatwaves demonstrate the high priority given to heat protection in Hesse [13,24]. However, a large-scale study from Germany revealed that only a minority of staff consider heat protection measures to be adequate [25]. Other climate adaptation measures, such as protection against storms or flooding, are not a major consideration, although a significant proportion of facilities are located in regions at risk. These findings are also consistent with the results reported in the literature. Protection against cold spells, droughts, or storms is rarely, if ever, considered in hospital emergency plans [8]. Another recent German study concluded that awareness of climate adaptation among healthcare sector stakeholders is rather mixed [26].

Contrary to this hypothesis, no significant correlation was observed between provider size and the implementation of measures for climate mitigation or climate adaptation. Hypotheses H3 and H4 (H3: more beds, more measures; H4 larger provider, more measures) were therefore rejected. Rather, it does not appear that objective factors explain which facilities implement such measures. On the one hand, nationwide projects show that large facilities are often involved in such initiatives [10]. On the other hand, Litke et al. and Khosravi et al. reported that the individual awareness and knowledge of managers and employees in particular are decisive factors [23,26].

Even adjustments to staffing structures as part of climate adaptation, such as modifying nursing schedules, are rarely considered (confirmation of H5). A survey conducted by the German Hospital Institute revealed a similar situation. Only 6% of the hospitals are actively pursuing capacity increases, whereas a further 45% are not doing it at all. Early warning systems also barely play any role [8].

This study has strengths and limitations. With a 30% participation rate among nursing homes, it achieved a comparatively high response rate and offered initial insight into how facilities in the Marburg-Fulda region prepare for climate change. However, the limited time available for responses may have reduced their depth, and socially desirable answers or nonresponse bias could have influenced the results. Owing to the small, regionally homogeneous sample, the findings should only be generalized with caution.

Practical studies are necessary to develop targeted implementation tools for hospitals and nursing homes. Expanding the study region would also be valuable to gain further insights through a larger sample size.

## 5 Conclusion

Nursing homes and hospitals in the Marburg–Fulda region have implemented selected fundamental climate mitigation and adaptation measures, particularly low-threshold actions such as energy-saving practices. However, the implementation of additional structural and technical measures for climate mitigation and adaptation remains limited. Overall, the findings indicate that the current level of engagement is insufficient to achieve substantial climate resilience. Although the authorities in Hesse have initiated first steps, the status quo demonstrates that the healthcare system cannot yet be considered climate-resilient.

## CRediT authorship contribution statement

**Caroline Maria Körner:** Writing – original draft, Visualization, Validation, Project administration, Methodology, Investigation, Formal analysis, Data curation, Conceptualization. **Max Geraedts:** Writing – review & editing, Supervision, Conceptualization.

## Declaration of competing interest

The authors declare that they have no known competing financial interests or personal relationships that could have appeared to influence the work reported in this paper.

## Appendix Supplementary materials

Image, application 1

Image, application 2

Image, application 3

Image, application 4

## Footnotes

Supplementary material associated with this article can be found, in the online version, at doi:10.1016/j.joclim.2026.100685.
