# Supplementary material for: Assessing climate change preparedness in hospitals and nursing homes in Hesse, Germany
Source: J Clim Chang Health. 2026 Apr 14;29:100685. doi: 10.1016/j.joclim.2026.100685 (PMC13092870; doi:10.1016/j.joclim.2026.100685)
Supplement: Supplementary file 1 [file mmc1.pdf]

Survey Instrument - Hospitals

Nr.	Question	Possible responses	Examples
	Description of research project: HABITAT (Health Affected by Climate Change and Air Pollution – Pathophysiology and Regional Management) is a research consortium funded by the Hessian LOEWE program that deals with the health effects of weather events and climate change. In this context, we are interested in whether and to what extent the institution already engages in climate protection.		
	Consent to data processing: Data analysis is carried out on an institution-specific basis, i.e., characteristics of the institution (such as number of beds, operator, location) are also taken into account in the data analysis. The data is pseudonymized. The evaluations are carried out with reference to the facility-related characteristics, but it is no longer possible to identify the facility in question.		
1	Does your facility have written guidelines or strategies on climate mitigation?	yes/no	
2	Does your facility have written guidelines or strategies on climate protection?	yes/no	
3	Have you already taken measures for climate mitigation in your facility?	yes/no	
3.1.	If so, what climate mitigation measures are you implementing?	open-ended	Building energy, electricity efficiency, heat efficiency, renewable energies, medical products, (anesthetic) gases, nutrition
3.2	Have you already taken measures for climate adaptation in your facility?	yes/no	

3.3	If so, what measures are you implementing to adapt to the effects of climate change?	open-ended	Drainage during heavy rainfall (infiltration areas, water pumps), heat regulation (air conditioning, shading, green areas/blue areas), capacity increases for health consequences of climate change (staff, ventilators), early warning systems (heat waves, infectious diseases), staff awareness training
3.4	Do you use air conditioning to protect patients and staff from the heat?	yes/no	
4	Have you adapted supply structures (e.g. schedules) in your facility in order to respond to extreme weather events or climate change?	yes/no	
4.1.	If so, what adjustments were made?	open-ended	Increasing staffing levels, providing more equipment

5	Do you need to prepare a sustainability report in accordance with EU guidelines this year?	yes/no	Corporate Sustainability Reporting Directive (CSRD)
6	Do you have a climate protection manager or someone in a similar role at your organization?	yes/no	Alternative terms: Sustainability/Environmental Protection Officer
7	Do you make use of funding opportunities for climate protection or climate change adaptation measures?	yes/no	
8.1	What barriers do you see in the implementation of climate protection or climate change adaptation?	open-ended	High staff turnover, staff shortages, lack of resources (materials, etc.), building renovation/structural modifications, financial hurdles
8.2	What other forms of support (e.g., advice, financial assistance) would be helpful for your institution to better implement climate protection or climate change adaptation measures?	open-ended	
